# Prevalence and Associated Factors of Renal Disease in Saudi Residents Attending Primary Health Care Centers in Riyadh, Saudi Arabia: Cross-Sectional Study

**DOI:** 10.2196/81849

**Published:** 2026-03-26

**Authors:** Saad Alshahrani, Ashraf A El-Metwally, Awad Alshahrani, Badr F Al-Khateeb, Aljohrah Ibrahim Aldubikhi, Khadijah Angawi, Amani Alharthy, Lubna Alnaim, Amal Mousa Zaidan, Raed Aldahash

**Affiliations:** 1 Department of Surgery, Division of Urology, College of Medicine, Prince Sattam bin Abdulaziz University, Alkharj, Saudi Arabia; 2 College of Public Health & Health Informatics, King Saud bin Abdulaziz University for Health Science, KSAU-HS, Riyadh, Saudi Arabia, 966 59 480 0755; 3 King Abdullah International Medical Research Center, Riyadh, Riyadh Region, Saudi Arabia; 4 Ministry of National Guard-Health Affairs, Riyadh, Saudi Arabia; 5 College of Medicine, King Saud bin Abdulaziz University for Health Science, Riyadh, Saudi Arabia; 6 King Abdulaziz Medical City, Ministry of the National Guard-Health Affairs, Riyadh, Saudi Arabia; 7 College of Health Sciences, Saudi Electronic University, Riyadh, Saudi Arabia; 8 Department of Health Services and Hospital Administration, Faculty of Economics and Administration, King Abdulaziz University, Jeddah, Saudi Arabia; 9 Department of Health Science, College of Health and Rehabilitation Sciences, Princess Nourah bint Abdulrahman University, Riyadh, Saudi Arabia; 10 Department of Clinical Nutrition, College of Applied Medical Sciences, King Saud bin Abdulaziz University for Health Science, Al Ahsa, Saudi Arabia

**Keywords:** renal disease, prevalence, risk factors, Saudi Arabia, primary health care, PHC, associated factors

## Abstract

**Background:**

Renal disease represents a significant and growing public health concern globally and within Saudi Arabia. Despite the increasing burden of noncommunicable diseases, population-based data on the prevalence and determinants of renal disease in Saudi Arabia remain limited. Understanding epidemiology, including prevalence and associated risk factors of renal disease in the context of Saudi Arabia, is essential for designing preventive and early detection strategies.

**Objective:**

This study aims to estimate the prevalence of renal disease and to identify sociodemographic, behavioral, and clinically associated factors among adults attending primary health care centers (PHCs) in Riyadh, Saudi Arabia.

**Methods:**

A cross-sectional, community-based study was conducted between March 2023 and July 2023 across 48 PHCs within Riyadh, using a consecutive sampling strategy. Adults aged 18 years and older were recruited and completed a validated, interviewer-administered electronic questionnaire assessing sociodemographic characteristics, lifestyle behaviors, and medical history, including comorbid conditions and health care access. Multivariable logistic regression was used to determine factors associated with self-reported renal disease, with results expressed as adjusted odds ratios (AORs) and 95% CIs, ensuring model adequacy and precision.

**Results:**

A total of 14,239 participants were surveyed (n=7519, 52.8% female; mean age 41.6, SD 13.2 y). The prevalence of self-reported renal disease (including chronic kidney disease and kidney stones) was 3.5% (n=504). Individuals aged younger than 50 years had higher odds of renal disease (AOR 1.22, 95% CI 1.02‐1.47). Female individuals were more likely to report renal disease than males (AOR 1.51, 95% CI 1.24‐1.84). Participants with health insurance had increased odds (AOR1.74; 95% CI 1.44‐2.11). Smoking was strongly associated with renal disease (AOR 3.07, 95% CI 2.38‐3.96). Other important associated factors included comorbidities such as diabetes (AOR 1.51, 95% CI 1.12‐2.04), hypertension (AOR 2.27, 95% CI 1.67‐3.08), obesity (AOR 12.75, 95% CI 9.97‐16.30), hypercholesterolemia (AOR 1.93, 95% CI 1.43‐2.60), and heart disease (AOR 6.84, 95% CI 5.14‐9.10).

**Conclusions:**

This large, community-based cross-sectional study identifies a significant burden of renal disease among adults in Riyadh and highlights several modifiable risk factors that could be targeted in preventive health programs. The findings emphasize the importance of integrating renal health screening within PHCs and strengthening public health strategies addressing obesity, hypertension, and smoking. While the study relied on self-reported data without biomarker or clinical confirmation, potentially underestimating true prevalence and introducing misclassification bias, it provides a crucial population-level baseline that can guide resource allocation and inform the development of nationwide surveillance systems for early detection of renal disease.

## Introduction

Renal disease represents a significant and growing challenge for health care systems worldwide [1-3]. In its early stages, kidney disease is often asymptomatic, progressing silently until substantial renal function is lost [4]. Without timely detection and management, it may advance to end-stage renal disease, necessitating dialysis or kidney transplantation [5,6]. Traditionally associated with older populations, renal dysfunction increasingly affects younger age groups due to rising rates of diabetes mellitus, hypertension, and obesity, conditions particularly prevalent in Middle Eastern populations [7-9]. Recent global estimates from the Global Burden of Disease Study highlight chronic kidney disease (CKD) among the top 10 causes of mortality worldwide, reflecting a steady increase in prevalence and disability-adjusted life years over the past decade [10,11]. Emerging studies also emphasize the link between metabolic syndrome, cardiovascular disease, and renal dysfunction, reinforcing shared risk factor pathways for CKD progression [12-14].

Saudi Arabia is undergoing rapid demographic, nutritional, and lifestyle transitions that have contributed to the rising burden of noncommunicable diseases, including diabetes and cardiovascular disorders, with downstream effects on renal health [15-17]. Despite expanding tertiary care services, early identification and risk stratification of individuals at increased risk of kidney disease remain limited [18-20]. National reports indicate rising prevalence of CKD and associated risk factors, with projections suggesting substantial increases in the demand for renal replacement therapies over the next decade [21,22]. Previous research has mainly focused on high-risk groups such as patients with diabetes, those on dialysis, or individuals already diagnosed with CKD [23-25]. Population-based data remains scarce, and existing studies show heterogeneity in methodologies, diagnostic criteria, and definitions of kidney dysfunction, limiting comparability across regions and subpopulations [26-28]. Furthermore, few studies have investigated lifestyle and behavioral determinants, such as physical inactivity, dietary patterns, and water consumption that may influence kidney health [29,30].

A critical gap remains in understanding the factors associated with renal disease across diverse demographic and clinical backgrounds in Saudi Arabia. Identifying these factors is essential for targeted interventions, national screening strategies, and efficient health care resource allocation [31]. Health transformation emphasizes preventive health care, precision public health, and early detection programs for chronic diseases, highlighting the importance of timely epidemiological insights into renal disease [31-33]. In this context, this study aims to examine both the prevalence and associated factors of renal disease among adults attending primary health care centers (PHCs) in Riyadh, Saudi Arabia. By identifying demographic, clinical, and lifestyle factors associated with renal dysfunction, this study seeks to provide evidence to inform public health policy, guide clinical practice, and support national prevention programs. Ultimately, our objective is to generate a clearer understanding of the epidemiological landscape of kidney disease in Saudi Arabia and to provide a foundation for future longitudinal and interventional research.

## Methods

### Study Design, Duration, Setting, and Sampling Strategy

A cross-sectional study was conducted between March 2023 and July 2023 across PHCs in Riyadh, Saudi Arabia. The survey included adult Saudi residents aged 18 years and older who attended PHCs during the study period. To minimize selection bias, consecutive sampling was used whereby all eligible individuals attending the PHCs during the data collection period were invited to participate until the required sample size was achieved. Data collection took place in the outpatient waiting areas of participating PHCs. Trained data collectors approached eligible individuals, explained the purpose of the study, obtained written informed consent, and administered a structured, interviewer-guided electronic questionnaire. The study period spanned from March 1, 2023, to July 31, 2023. This recruitment approach allowed inclusion of a broad and diverse population of PHC attendees across Riyadh, enhancing the representativeness and external validity of the findings. The reporting of this research study adheres to the STROBE (Strengthening the Reporting of Observational Studies in Epidemiology) guidelines for cross-sectional studies to ensure methodological transparency and completeness of reporting [34]. The completed STROBE checklist is provided as a supplementary file (Checklist 1).

### Eligibility Criteria and Study Participants

The study included adult individuals aged 18 years and older who attended selected PHCs in Riyadh during the data collection period. Eligibility was not restricted by nationality or residency status; both Saudi and non-Saudi residents were invited to participate. Exclusion criteria included individuals aged younger than 18 years and PHC staff or employees. Eligible individuals were identified on-site and invited to participate by trained data collectors. Those who declined to provide written informed consent were excluded.

### Sample Size

A total of 14,239 participants completed the survey and were included in the final analysis. The sample size was determined using the single population proportion formula, assuming a 50% estimated prevalence of renal disease (for maximum variability), a 95% confidence level, and a 2% margin of error. The minimum required sample was 2,401; however, a substantially larger sample was achieved to enhance precision and generalizability across subgroups.

### Study Questionnaire Development, Adaptation, and Validation

#### Study Questionnaire Development

The survey instrument used in this study was developed to comprehensively assess health-related behaviors, medical history, and sociodemographic characteristics among adults attending PHCs in Riyadh. The use of previously validated survey items, expert review, pilot testing, and reliability assessment was intended to minimize measurement bias and enhance the accuracy and consistency of self-reported data.

#### Adaptation and Validation

The questionnaire was primarily adapted from existing validated instruments used in population health surveys internationally, ensuring relevance, comparability, and methodological rigor [35-37]. Additional items were included to capture specific national health care priorities and contextual factors relevant to Saudi Arabia. This approach ensured that the tool combined validated measures with region-specific questions to reflect the study objectives accurately.

#### Translation and Cultural Adaptation

The original English version of the questionnaire underwent a forward translation into Arabic, followed by back-translation into English by independent bilingual experts to confirm semantic equivalence and cultural appropriateness. This process ensured that both language versions conveyed the intended meaning consistently and were suitable for the target population.

#### Content and Face Validity

Content validity was evaluated by a panel of 15 subject matter experts, including clinicians and public health professionals from multiple regions of Saudi Arabia including nephrologists, urologists, primary care physicians, endocrinologists, cardiologists, and public health professionals. These experts evaluated the questionnaire for clarity, relevance, and suitability, ensuring that items adequately captured factors pertinent to renal health, lifestyle behaviors, and comorbid conditions. Based on their recommendations, several items were refined or removed to improve the instrument’s precision and effectiveness. Experts also assessed each item for relevance, clarity, and appropriateness within the national context. Following content validation, the Arabic version of the questionnaire was pilot-tested for face validity among 200 participants to assess clarity, comprehensibility, and overall acceptability. Feedback from pilot participants informed minor revisions to wording and phrasing, enhancing readability and interpretability.

#### Reliability Assessment

To evaluate consistency, a test-retest reliability assessment was conducted with 100 pilot participants who completed the questionnaire on 2 separate occasions. The second administration was conducted over the phone after minor refinements were made based on participant feedback. The resulting test-retest reliability coefficient of 0.83 demonstrated strong consistency over time.

#### Pilot Testing and Justification for Site Selection

The pilot study was conducted in Hail City, which was selected by the Central Health Services Reform Management Team due to its demographic and health characteristics being broadly comparable to those of the Riyadh population. Hail’s population structure, health care use patterns, and prevalence of major chronic diseases closely mirror those of urban regions such as Riyadh, making it an appropriate and representative site for pretesting the instrument. A total of 100 patients and 20 focus group participants provided feedback on the clarity, comprehensibility, and cultural relevance of the questionnaire. Based on their input, minor revisions were implemented to enhance wording and ease of interpretation. The finalized questionnaire, following validation and pilot testing, was deemed ready for implementation across 48 PHCs in Riyadh.

### Data Collection Procedures at PHCs

Data collection was conducted through an interviewer-assisted electronic survey. To reduce information and interviewer bias, data collectors received standardized training, and a structured, interviewer-guided electronic questionnaire was used to ensure uniform administration across all sites. Trained data collectors stationed at PHCs in Riyadh, Saudi Arabia, administered the digital questionnaire using handheld devices such as iPads or Android tablets. Prior to initiating the survey, each data collector verified the eligibility of individuals present at the PHCs, confirming that participants were aged 18 years or older. Eligible individuals were then approached, provided with a clear explanation of the study’s purpose and procedures, and asked to give written informed consent. Participation in the survey was entirely voluntary. Those who agreed to participate completed the questionnaire under the guidance of the data collectors, who were available to clarify questions and ensure accurate responses.

The survey instrument captured a wide range of variables. Sociodemographic information included age, sex, marital status, household size, educational attainment, employment status, and self-rated health. Additionally, the questionnaire explored lifestyle and behavioral factors such as smoking habits, fast food consumption, alcohol use, and levels of physical activity or exercise. Participants were also asked to report any known health conditions, including hypertension, diabetes, obesity, and hypercholesterolemia. In total, 14,239 individuals completed the survey and were included in the final dataset for analysis.

### Assessments and Data Sources

#### Outcome Variable

The primary outcome was self-reported renal disease (yes or no), determined based on participants’ responses to the question: “Have you ever been told by a physician that you have any kidney-related condition (such as kidney stones, infection, or chronic kidney disease)?” Responses were coded dichotomously (yes=1; no=0). The outcome, therefore, reflects any type of renal disease diagnosed by a physician, encompassing both medical and surgical conditions. No laboratory confirmation (eg, serum creatinine, estimated glomerular filtration rate [eGFR], and urinalysis) was obtained as part of this study, consistent with the survey-based design. Recall bias was mitigated by limiting outcome assessment to physician-diagnosed conditions and by administering the questionnaire in a supervised setting with trained interviewers available for clarification.

#### Independent Variables

Independent variables included sociodemographic characteristics, such as age, sex, marital status, educational attainment, employment status, nationality, and health insurance coverage, as well as clinical factors, including the presence of participants’ self-reported physician diagnoses such as diabetes, hypertension, obesity, cardiovascular disease, and hyperlipidemia. Age was categorized into 2 groups (<50 and ≥50 y) based on clinical and epidemiological considerations. Evidence from renal disease research indicates that the risk of kidney dysfunction increases substantially after middle age, largely due to cumulative exposure to chronic conditions such as hypertension, diabetes, and cardiovascular disease. This categorization allowed differentiation between younger adults with potential early-onset or lifestyle-related renal risk and older adults at higher risk of age-related and comorbidity-associated renal disease. The selected cutoff is consistent with those used in previous regional and international studies, facilitating comparability and public health interpretation. Lifestyle factors were also assessed, encompassing smoking, dietary habits (eg, fast-food consumption), and physical activity. Perceived health status was measured using a 5-point Likert scale, with the response options scored as follows: 0 (“poor”), 1 (“fair”), 2 (“good”), 3 (“very good”), and 4 (“excellent”).

#### Data Sources

All data were obtained through a structured, interviewer-administered electronic questionnaire. The tool was developed by the Central Health Services Reform Management Team in collaboration with regional experts as part of a national health assessment initiative. The survey collected self-reported information only, and no clinical or laboratory assessments were performed. There were no missing data in this study, as the electronic data collection system was designed to require completion of all questionnaire items before submission.

### Statistical Analysis Approach

The distribution of all variables was initially examined using histograms to assess normality. Descriptive statistics were then used to summarize the data. For continuous variables that followed normal distributions, such as age and results were expressed as means and SDs. To facilitate further analysis, age was also categorized into distinct groups. Categorical variables, including insurance status, perceived health, marital status, employment status, and educational level, were summarized using frequencies and percentages. Given that the main outcome variable, renal disease, was dichotomous (yes or no), binary logistic regression was used for analysis. Initially, univariate logistic regression was conducted to explore associations between each independent variable and the likelihood of having renal disease. Variables demonstrating a *P* value less than 0.25 in the univariate analysis were selected for inclusion in the multivariable logistic regression model. The multivariable logistic regression was then carried out to identify factors associated independently with renal disease, with statistical significance set at *P*<.05.

To enhance model interpretability and stability, a parsimonious modeling strategy was adopted to reduce the risk of overfitting and potential multicollinearity, given the relatively large number of predictors in relation to the number of outcome events. Accordingly, 2 separate multivariable logistic regression models were constructed. The first model included sociodemographic variables, while the second model examined behavioral and comorbidity-related factors. The behavioral or comorbidity model was adjusted for key demographic covariates, specifically age and sex, to account for major confounding effects while preserving model robustness.

Results from the multivariable analyses were reported as adjusted odds ratios (AORs) with corresponding 95% CIs, and statistical significance was set at *P*<.05. All statistical analyses were performed using IBM SPSS Statistics for Windows (version 26).

### Ethical Considerations

The study was conducted in accordance with the principles outlined in the Declaration of Helsinki and received approval from the Institutional Review Board of King Fahad Medical City, Riyadh (protocol code: 22-397E; approval date: October 6, 2022). Written informed consent was obtained from all participants prior to data collection, and participants were informed of their right to withdraw from the study at any stage without any consequences. Data were collected anonymously, and no personally identifiable information was recorded to ensure privacy and confidentiality. All electronic data were stored on password-protected devices accessible only to authorized research personnel. Participants did not receive any financial or nonfinancial compensation for their involvement in the study. Furthermore, no images or identifiable participant data were collected or included in this paper or its supplementary materials.

## Results

### Sociodemographic Profile of Surveyed Saudi Residents

The sociodemographic profile of individuals who participated in the survey at PHCs in Riyadh is detailed in Table 1. The average age of the surveyed population was approximately 59.8 (SD 16.4) years. The largest age bracket comprised individuals aged 50 years or older, representing 66% (n=9391) of the participants, and most of the surveyed individuals were married (n=9300, 65.3%). Regarding educational attainment, 31.7% (n=4509) had attained less than a college education, whereas 51.4% (n=7317) were employed. A significant proportion of participants perceived their health as very good (n=5076, 35.6%) or excellent (n=4798, 33.7%). The majority of the participants (n=10782, 75.7%) indicated a lack of insurance coverage. The prevalence of smoking was 27.7% (n=3942). In terms of physical activity, a smaller majority (n=8641, 60.7%) reported engaging in exercise. The prevalence of obesity was 5.2% (n=737), whereas the prevalence of diabetes, hypertension, and renal disease was 12.4% (n=1765), 11.1% (n=1580), and 3.5% (n=504), respectively.

**Table 1.** Sociodemographic characteristics of Saudi residents (N=14,239) attending primary health care centers in Riyadh, Saudi Arabia, from March 2023 to July 2023: findings from a cross-sectional study on renal disease prevalence and associated factors.

Variable	Value, n (%)
Age (y)	
< 50	4848 (34.0)
≥50	9391 (66.0)
Education attainment	
Less than a college education	4509 (31.7)
At least a college education	9730 (68.3)
Biological sex	
Female	8062 (56.6)
Male	6177 (43.4)
Marital status	
Not married	4939 (34.7)
Married	9300 (65.3)
Employment	
Employed	7317 (51.4)
Unemployed	6922 (48.6)
Perceived health status	
Excellent	4798 (33.7)
Very good	5076 (35.6)
Good	2815 (19.8)
Fair	1256 (8.8)
Poor	294 (2.1)
Insurance coverage	
Yes	3457 (24.3)
No	10,782 (75.7)
Smoking status	
No	10,297 (72.3)
Yes	3942 (27.7)
Exercise	
No	5598 (39.3)
Yes	8641 (60.7)
Obesity	
No	13,502 (94.8)
Yes	737 (5.2)
Diabetes	
No	12,474 (87.6)
Yes	1765 (12.4)
Hypertension	
No	12,659 (88.9)
Yes	1580 (11.1)
Hypercholesterolemia	
No	13,386 (89.4)
Yes	853 (10.6)
Heart disease	
No	13,845 (95.1)
Yes	394 (4.9)
Renal disease	
No	13,735 (96.5)
Yes	504 (3.5)

### Sociodemographic Factors Associated With Renal Disease: Univariate and Multivariable Analysis

#### Univariate Analysis

Table 2 presents the univariable analysis exploring the relationship between various sociodemographic factors and the presence of renal disease among Saudi residents who participated in the survey. Age was significantly associated with renal disease (*P*=.03). Compared to individuals aged over 50 years, those aged less than 50 years had higher odds of renal disease (OR 1.26, 95% CI 1.05‐1.52). The relatively narrow CI indicates a moderately precise estimate, suggesting that younger participants consistently had greater odds of renal disease across the sample. Sex showed a significant association (*P*<.001), with female individuals having 1.58 times higher odds of renal disease compared to male individuals (OR 1.58, 95% CI 1.31‐1.91). This estimate is precise, as reflected by the narrow CI, indicating a robust relationship between sex and renal disease. Compared to those with less than a college education, individuals with at least a college education had slightly higher odds (OR 1.15, 95% CI 0.95‐1.40) of renal disease. However, the CI includes 1.0, suggesting that the association lacks statistical precision and may not be clinically meaningful. Employment status showed a significant association, with unemployed individuals having 1.20 times higher odds of renal disease compared to employed individuals (OR 1.20, 95% CI 1.00‐1.43). The CI borders unity, indicating limited precision and a possible weak association. Finally, individuals having insurance coverage exhibited 1.71 times higher odds of renal disease compared to those without insurance (OR 1.71, 95% CI 1.42‐2.06). The narrow CI suggests a highly precise and consistent estimate.

**Table 2.** Sociodemographic factors associated with renal disease among Saudi residents attending primary health care centers in Riyadh, Saudi Arabia, from March 2023 to July 2023: results from univariate and multivariable logistic regression analyses in a cross-sectional study.

Associated factors	Univariable analysis				Multivariable analysis			
	OR^a^ (95% CI)			*P* value	AOR^b^ (95% CI)			*P* value
Age (y) (reference: ≥50)								
<50	1.26 (1.05-1.52)			.01	1.22 (1.02-1.47)			.03
Sex (reference: male)								
Female	1.58 (1.31-1.91)			<.001	1.51 (1.24-1.84)			<.001^c^
Education (reference: less than a college education)								
At least a college education	1.15 (0.95-1.4)			.16	1.16 (0.95-1.43)			.15
Marital status (reference: single)								
Married	0.98 (0.81-1.18)			.83	—^d^			—
Employment status (reference: employed)								
Unemployed	1.2 (1-1.43)			.04	1.16 (0.96-1.42)			.13
Insurance coverage (reference: no)								
Yes	1.71 (1.42-2.06)			<.001	1.74 (1.44-2.11)			<.001^c^

a OR: odds ratio.

b AOR: adjusted odds ratio, which was calculated after controlling for sociodemographic factors that were significant at *P*<0.25 in univariate analysis

c *P*<.05 was considered significant in multivariable analysis.

d not applicable.

#### Multivariable Analysis

After adjusting for the other factors, age was found to be a significant factor for renal disease. Compared to individuals aged over 50 years, those aged less than 50 years had higher odds of renal disease (AOR 1.22, 95% CI 1.02‐1.47). Sex was also significantly associated with renal disease, with female individuals having 1.51 times higher AOR compared to male individuals (AOR 1.51, 95% CI 1.24‐1.84). This narrow CI demonstrates high precision and strengthens confidence in the observed sex difference. Insurance coverage remained significantly associated with renal disease, with individuals having insurance coverage exhibiting 1.74 times higher AOR compared to those without insurance (AOR 1.74, 95% CI 1.44‐2.11). The tight CI further supports the precision and consistency of this association. Education and employment status did not retain statistical significance in the multivariable model as shown in Table 2.

### Behavioral Risk Factors and Comorbidities Associated With Renal Disease: Univariate and Multivariable Analysis

#### Univariate Analysis

Individuals who reported smoking had 5.22 times higher odds of having renal disease compared to non-smokers (OR 5.22, 95% CI 4.33‐6.29). The narrow CI indicates a highly precise and consistent estimate. Participants with diabetes had 5.12 times higher odds of renal disease compared to those without diabetes (OR 5.12, 95% CI 4.25‐6.16), demonstrating good precision and suggesting a strong, stable association. The presence of hypertension was associated with 10.11 times higher odds of renal disease (OR 10.11, 95% CI 8.41‐12.15). The relatively narrow CI supports the reliability of this association. Obese individuals had a substantially higher odds of renal disease at 38.64 times compared to nonobese individuals (OR 38.64, 95% CI 31.65‐47.18). Although the CI range is wider than others, it remains far from unity, reflecting a strong and highly significant relationship. Hypercholesterolemia was associated with 14.83 times higher odds of renal disease (OR 14.83, 95% CI 12.30‐17.87), indicating a consistent and precise association. Finally, individuals with heart disease had a remarkably higher odds of renal disease at 40.69 times compared to those without heart disease (OR 40.69, 95% CI 33.12‐49.99). Despite a wider CI, the estimate remains highly precise due to its magnitude and narrow relative interval.

#### Multivariable Analysis

After adjusting for the other variables in the model, smoking remained significantly associated with renal disease (*P*<.001), with smokers having 3.07 times higher AOR of renal disease compared to nonsmokers (AOR 3.07, 95% CI 2.38‐3.96). The narrow CI reflects a precise estimate, confirming the robustness of the association. Diabetes remained significantly associated with renal disease, with individuals with diabetes having 1.51 times higher adjusted odds of renal disease (AOR 1.51, 95% CI 1.12‐2.04) compared to those without diabetes. The CI does not cross 1.0, indicating a statistically meaningful and fairly precise association. Hypertension remained significantly associated with renal disease (*P*<.001), with individuals with hypertension having 2.27 times higher adjusted odds of renal disease (AOR 2.27, 95% CI 1.67‐3.08) compared to those without hypertension. The narrow CI suggests precision and reinforces the strength of this relationship. Obesity remained strongly associated with renal disease (*P*<.001), with obese individuals having 12.75 times higher adjusted odds of renal disease (AOR 12.75, 95% CI 9.97‐16.30) compared to nonobese individuals. Although the CI is somewhat wide, the lower bound is well above 1, indicating a very strong and precise association. Hypercholesterolemia remained significantly associated with renal disease (*P*<.001), with individuals having hypercholesterolemia exhibiting 1.93 times higher adjusted odds of renal disease (AOR 1.93, 95% CI 1.43‐2.60) compared to those without hypercholesterolemia. The relatively narrow CI indicates good precision and reliability. Finally, heart disease remained significantly associated with renal disease (*P*<.001), with individuals reporting heart disease exhibiting 6.84 times higher adjusted odds of renal disease (AOR 6.84, 95% CI 5.14‐9.10), as shown in Table 3. The narrow CI demonstrates strong precision and reinforces the robustness of this association.

**Table 3.** Behavioral risk factors and comorbidities associated with renal disease among Saudi residents attending primary health care centers in Riyadh, Saudi Arabia, from March 2023 to July 2023: univariate and multivariable logistic regression findings from a cross-sectional study.

Associated factors	Univariate analysis		Multivariable analysis	
	OR^a^ (95% CI)	*P* value^b^	AOR^c^ (95% CI)	*P* value^d^
Smoking (reference: no)				
Yes	5.22 (4.33-6.29)	<.001	3.07 (2.38-3.96)	<.001
Diabetes (reference: no)				
Yes	5.12 (4.25-6.16)	<.001	1.51 (1.12-2.04)	<.001
Hypertension (reference: no)				
Yes	10.11 (8.41-12.15)	<.001	2.27 (1.67-3.08)	<.001
Obesity (reference: no)				
Yes	38.64 (31.65-47.18)	<.001	12.75 (9.97-16.3)	<.001
Hypercholesterolemia (reference: no)				
Yes	14.83 (12.3-17.87)	<.001	1.93 (1.43-2.6)	<.001
Heart disease (reference: no)				
Yes	40.69 (33.12-49.99)	<.001	6.84 (5.14-9.1)	<.001

a OR: odds ratio.

b *P* value of <.25 was considered significant in univariable analysis.

c AOR: adjusted odds ratio, which was calculated in the multivariable analysis after controlling for sex, age, and mutual adjustment with other behavioral risk factors and comorbidities.

d *P* value of <.05 was considered significant in multivariable analysis.

## Discussion

### Principal Findings

This study aimed to investigate the prevalence and associated factors of self-reported, physician-diagnosed renal disease among adults attending PHCs in Riyadh, Saudi Arabia. Renal disease in this study encompassed both medical and surgical conditions, as reported by participants, without laboratory confirmation of diagnosis. The findings revealed a self-reported renal disease prevalence of 3.5% (n=504), which reflects disease awareness rather than actual prevalence, given that diagnoses were based solely on self-report. Multiple demographic, behavioral, and clinical factors, including age, sex, insurance coverage, smoking, diabetes, hypertension, obesity, hypercholesterolemia, and heart disease, were independently associated with higher odds of renal disease. These findings underscore the multifactorial nature of renal pathology and provide valuable insights into population-level risk determinants that can inform public health and clinical strategies in Saudi Arabia.

The observed prevalence of 3.5% (n=504) reflects the burden within a primary care-attending population, consistent with reports from other regional studies. While direct comparisons are complicated by differences in diagnostic criteria and population characteristics [26-28], this prevalence aligns with previous estimates indicating a notable burden of kidney dysfunction ranging between 4.76% to 10% [29,38,39]. Although comparable, this prevalence likely under-represents the true magnitude of renal disease, the outcome measure relied entirely on self-report, and it likely underestimates true disease prevalence and instead reflects participants’ awareness of having a renal condition. The absence of objective biomarkers such as serum creatinine, eGFR, or urinalysis limits diagnostic precision and may have led to both under- and over-reporting. This limitation emphasizes the need for population-based screening incorporating objective measures to capture the full spectrum of renal impairment within the community.

An unexpected finding was that individuals aged younger than 50 years reported higher odds of renal disease compared to those over 50 years. This result diverges from conventional epidemiological evidence, where older age is typically associated with higher CKD risk due to cumulative exposure to risk factors and age-related decline in renal function [40,41]. A plausible explanation is that younger, more educated, and health-conscious individuals are more likely to recognize and report a previous renal diagnosis, leading to differential awareness rather than a true shift in disease burden. Additionally, this pattern may reflect the contribution of renal stones, conditions more common among younger and middle-aged adults in hot climates like Riyadh, which could explain much of this association. These findings highlight the importance of distinguishing between chronic and acute, as well as surgical renal conditions in future studies.

Female individuals were found to have a significantly higher likelihood of renal disease compared to male individuals. This aligns with prior studies suggesting that women may be at increased risk due to biological differences, including hormonal influences on kidney function, higher tendency to develop kidney stones, or sociocultural factors influencing health care−seeking behavior and disease reporting [39,42-44]. This finding also highlights the importance of gender-sensitive approaches to renal health promotion and targeted screening for women at risk.

Participants with health insurance exhibited higher odds of renal disease. This is likely attributable to improved health care access and diagnostic opportunities among insured individuals, leading to higher detection and reporting rates rather than increased biological risk [45-47]. This finding supports the importance of health care access in promoting disease recognition and timely intervention.

Smoking was strongly associated with renal disease, with smokers having over 3 times the odds compared to nonsmokers. This is consistent with a robust body of evidence linking tobacco to kidney damage through mechanisms including increased oxidative stress, inflammation, and vascular injury [48-50]. Smoking accelerates the progression of both diabetic and hypertensive nephropathy and is a modifiable risk factor that should be a primary focus of prevention campaigns [48,49]. The high odds ratio observed underscores the critical need for integrating smoking cessation programs within renal disease prevention efforts in Saudi Arabia.

Several chronic health conditions were significantly associated with renal disease in this study, consistent with global literature. Individuals with diabetes had 1.5 times higher odds of renal disease, reaffirming diabetes as a leading cause of chronic kidney disease worldwide as found in other similar studies [43,51]. Poor glycemic control contributes to microvascular damage in the kidneys, leading to diabetic nephropathy [52]. Given the high prevalence of diabetes in Saudi Arabia, intensified efforts toward diabetes management and renal monitoring are essential. Consistent with other studies, hypertension was also associated with renal disease [39,42,43]. Hypertension contributes to kidney damage via increased glomerular pressure and vascular remodeling. The bidirectional relationship between hypertension and renal disease calls for integrated management approaches. However, as our outcome combined multiple renal conditions, these associations may reflect general comorbidity clustering or increased diagnostic attention among individuals with multiple chronic diseases.

As found in previous literature, obesity was strongly associated with renal disease indicating its substantial role in kidney disease risk [43]. Obesity induces renal hyperfiltration and promotes inflammation, hypertension, and diabetes, all of which contribute to renal impairment. This finding is particularly relevant given rising obesity rates in Saudi Arabia and suggests that weight management should be a cornerstone of kidney disease prevention. Individuals with high cholesterol levels had nearly twice the odds of renal disease as found in other studies [53,54]. Dyslipidemia can contribute to renal damage through atherosclerotic changes and glomerular injury, supporting the importance of lipid control in at-risk populations [55]. The presence of heart disease was strongly associated with renal disease, increasing the odds nearly 7-fold. This relationship reflects the cardiorenal syndrome, where heart and kidney dysfunction coexist and exacerbate each other [56]. Patients with cardiovascular disease require careful renal monitoring and vice versa. Moreover, the identified associated factors largely concur with international studies emphasizing the interplay between metabolic, cardiovascular, and behavioral risk factors in renal disease pathogenesis. Similar findings in Middle Eastern cohorts have reported obesity, hypertension, diabetes, and smoking as key drivers of kidney disease [57,58].

Overall, the findings should be interpreted as associations with self-reported awareness of kidney-related diagnoses rather than confirmed clinical disease. The study highlights the importance of health literacy, access to care, and lifestyle risk factors in shaping population awareness of renal health in Saudi Arabia.

### Strengths and Limitations

One of the major strengths of this study lies in its robust sampling methodology with a large and representative sample of individuals accessing primary health care services across Riyadh’s diverse urban and suburban populations. This sampling strategy enhances the generalizability of the findings within the region and provides a solid basis for informing regional health care planning and policy development. Additionally, the study benefits from comprehensive data collection encompassing a wide range of sociodemographic, behavioral, and clinical variables. The rigorous validation and reliability assessment of the survey instrument ensured high-quality data, strengthening the internal validity of the study. The application of multivariable logistic regression allowed for the identification of independently associated factors of renal disease, providing nuanced insights into the complex interplay of risk factors in this population.

Despite its strengths, several limitations should be noted. The cross-sectional design precludes causal inference and limits understanding of temporal relationships between exposures and renal disease. First, the definition of “renal disease” in this study was based on self-reported, physician-diagnosed conditions without specifying subtypes (eg, chronic kidney disease, renal stones, or infections), introducing potential ambiguity in interpretation. Second, the absence of laboratory or imaging confirmation (eg, serum creatinine, eGFR, and urinalysis) limits diagnostic accuracy and may have resulted in both underestimation and misclassification of true disease prevalence. Third, reliance on self-reported data introduces the possibility of recall and reporting bias, as participants’ awareness and understanding of their renal condition may vary by demographic and educational background. Fourth, the sample’s relatively high mean age may reflect a degree of sampling bias, as older individuals are more likely to attend PHCs, potentially influencing prevalence estimates. Although these limitations are acknowledged, reliance on self-report also reflects real-world challenges in laboratory-based surveillance within many PHC settings. Some observed ORs, particularly for obesity, are notably high and may reflect residual confounding from unmeasured factors such as diet, physical activity, socioeconomic status, or genetic predisposition. Nonetheless, the findings remain highly relevant for public health planning, providing the first large-scale population estimate of self-reported renal disease in Riyadh and serving as a benchmark for future screening and surveillance efforts that incorporate objective clinical measures.

### Policy Implications

While this study provides valuable insight into self-reported renal disease among PHC attendees in Riyadh, the findings should be interpreted cautiously given the reliance on self-reported, unverified diagnoses. Rather than implying a true epidemiologic shift, the observed associations, particularly the higher prevalence among younger, insured, and female participants, may reflect greater health awareness, reporting behavior, and access to health care services rather than genuine differences in disease burden. Accordingly, these findings highlight important awareness and surveillance gaps rather than immediate policy directives. Strengthening national health surveillance systems that integrate laboratory-based diagnostic confirmation (eg, eGFR and urinalysis) would allow more accurate estimation of kidney disease prevalence and its risk factors. Nevertheless, the study underscores the continued importance of public health strategies targeting modifiable risk factors, such as obesity, diabetes, hypertension, and smoking. Integrating kidney health into broader non-communicable disease prevention programs, alongside improved health literacy and early detection at the PHC level, could substantially improve renal health outcomes. Enhanced data collection infrastructure and population-based screening programs would further enable policymakers to design evidence-informed interventions and track progress toward national health goals.

### Conclusions

This study offers valuable insight into the self-reported prevalence and associated factors of renal disease among individuals attending PHCs in Riyadh, Saudi Arabia. While the findings indicate that a notable proportion of participants reported a prior physician diagnosis of kidney-related conditions, the reliance on self-reported data without laboratory confirmation suggests that these results more accurately reflect awareness and detection patterns rather than the true epidemiological burden of renal disease. Several modifiable factors, including smoking, obesity, hypertension, diabetes, and hypercholesterolemia, were found to be significantly associated with self-reported renal disease, consistent with established risk profiles in the literature. Demographic associations, such as those observed with age, sex, and insurance coverage, likely reflect differences in health care access and health awareness rather than causal relationships.

Overall, the study underscores the importance of strengthening surveillance systems and integrating laboratory-based screening within primary care to improve early identification and monitoring of kidney disease in Saudi Arabia. Future research using objective measures and longitudinal designs is needed to validate these associations and to better understand the true epidemiology of renal disorders in the population.

## Supplementary material

10.2196/81849 Checklist 1 STROBE checklist.

## Abbreviations

AOR: adjusted odds ratio
CKD: chronic kidney disease
eGFR: estimated glomerular filtration rate
OR: odds ratio
PHC: primary health care center
STROBE: Strengthening the Reporting of Observational Studies in Epidemiology
